# Traumatic right common femoral artery occlusion caused by blunt bicycle handlebar injury: a case report

**DOI:** 10.1186/s40792-019-0628-3

**Published:** 2019-04-22

**Authors:** Kenshi Yoshimura, Hirotsugu Hamamoto

**Affiliations:** Department of Cardiovascular Surgery, Almeida Memorial Hospital, 1509-2, Oaza Miyazaki, Oita city, Oita 870-1195 Japan

**Keywords:** Handlebar injury, Common femoral artery, Occlusion, Thromboendarterectomy

## Abstract

**Background:**

Traumatic femoral artery occlusion caused by blunt impact to the groin is rare; this condition is called the “motor-scooter handlebar syndrome.” We herein report a case of traumatic femoral artery occlusion and performed a literature review on its diagnosis and treatment.

**Case presentation:**

An 18-year-old man visited our hospital complaining of pain and swelling in his right groin and numbness in his right leg after a bicycle collision accident. Contrast computed tomography revealed an occlusion extending from the right external iliac artery to the common femoral artery. The right ankle–brachial index (ABI) was 0.50. We performed thrombectomy and femoral artery repair with a saphenous vein patch. The postoperative course was good, and the right ABI improved to 1.05.

**Conclusions:**

Motor-scooter handlebar syndrome is a rare complication of traumatic injury. The presence of vascular injury should be considered in patients with groin or lower abdomen injuries following an impact with handlebars or similar hard objects. This injury often needs surgical treatment; therefore, prompt diagnosis is the key to successful treatment.

## Background

Bone fractures caused by blunt trauma may be accompanied by vascular injury, and arterial occlusion may develop. Occlusion of the external iliac artery (EIA) or common femoral artery (CFA) following blunt trauma to the inguinal region by a bicycle or motorcycle handlebar without concomitant bone fracture is rare. This clinical condition was reported first as “motor-scooter handlebar syndrome” in 1968 [1]. Since then, some similar cases have been reported [2–28]. Nonetheless, this syndrome tends to be overlooked owing to its rarity and lack of awareness. Herein, we report a case of “motor-scooter handlebar syndrome” caused by a collision while riding a bicycle. The vascular injury was successfully treated by surgery. In addition, we have reviewed the literature.

## Case presentation

**Fig. 1:**
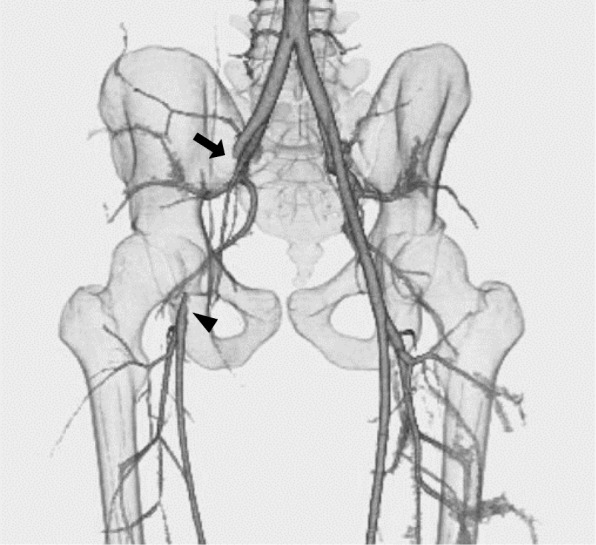
Preoperative CT angiography shows occlusion extending from the right EIA to CFA (arrow) and a contrast effect peripheral to the lesion from collateral circulation (arrowhead). CT, computed tomography; EIA, external iliac artery; CFA, common femoral artery

While riding a bicycle, an 18-year-old man (height, 165 cm; weight, 60.3 kg) collided with another bicycle coming from the left side. The right handlebar of his bicycle hit his groin. Although the numbness of the right lower limb that began immediately after the impact gradually improved, the patient was admitted to our hospital with right inguinal pain and swelling. Consistent with subcutaneous hematoma, the colors of the right and left leg were similar in the resting state; however, the right leg became pale after walking and he noticed mild claudication. There was no palpable pulse in the right pedal artery, but flow was recognized by pulse Doppler ultrasound. Contrast computed tomography (CT) for the evaluation of bone fracture or active bleeding revealed vascular occlusion extending from the right EIA to the CFA (Fig. 1). There was a contrast effect in the distal CFA just before the branching of the superficial and deep femoral arteries and the collateral circulation. A duplex scan showed no flow in the right EIA and small flow in the distal CFA. The right ankle–brachial index (ABI) was 0.50. Laboratory examination showed an elevated creatine kinase (CK) level of 1302 IU/L and slightly elevated glutamic oxaloacetic transaminase level of 43 IU/L. Glutamic pyruvic transaminase, lactate dehydrogenase (LDH), and potassium were normal with levels of 28 IU/L, 197 IU/L, and 4.5 mEq/L, respectively. With respect to inflammatory reaction, the white blood cell count and C-reactive protein level were slightly increased at 9630/μL and 0.87 mg/mL, respectively. The pathological state was similar to acute limb ischemia, and Rutherford classification was category I. However, the severity was not considered urgent as the Doppler detected pedal artery flow and the duplex scan detected CFA flow, both indicating that the blood flow to the right lower limb was maintained by collateral circulation. Although CK level was highly elevated, we did not consider emergent surgical treatment as other parameters were normal or only slightly elevated. Moreover, the patient hesitated the surgical treatment at that time. For these reasons, we decided to see the course for a while with conservative treatment, and heparin administration was started. The next day, contrast CT revealed the extension of the contrast effect in the EIA and no change in the obstruction of the CFA, whereas the duplex scan showed findings similar to that shown on the previous day. The lack of response to conservative treatment prompted surgical revascularization with thrombectomy and femoral artery repair, as well as patient’s surgical consent.

**Fig. 2:**
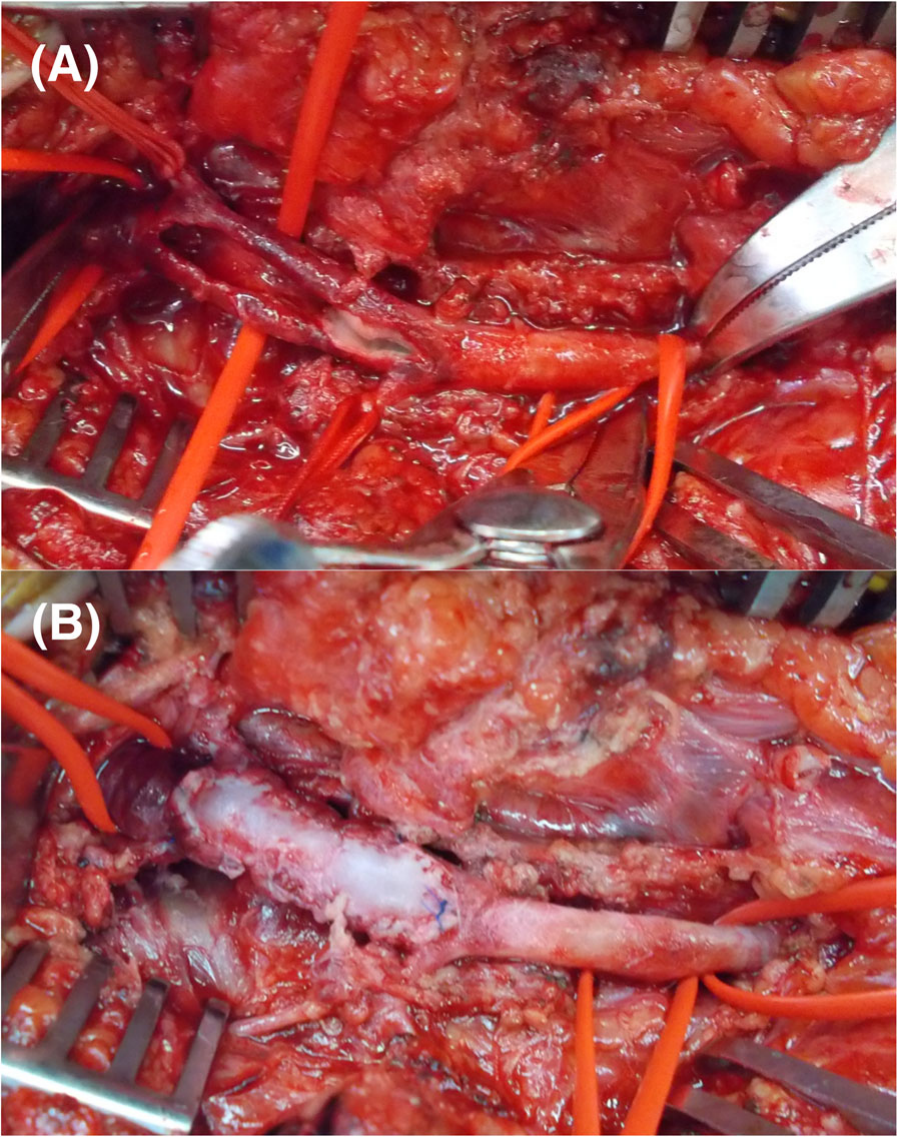
Intraoperative findings show that **a** the intima of the injured part almost disappeared and the dissected intima barely remained on the proximal and distal sides of the CFA. **b** The repair of the CFA was performed with a saphenous vein patch. CFA, common femoral artery

**Fig. 3:**
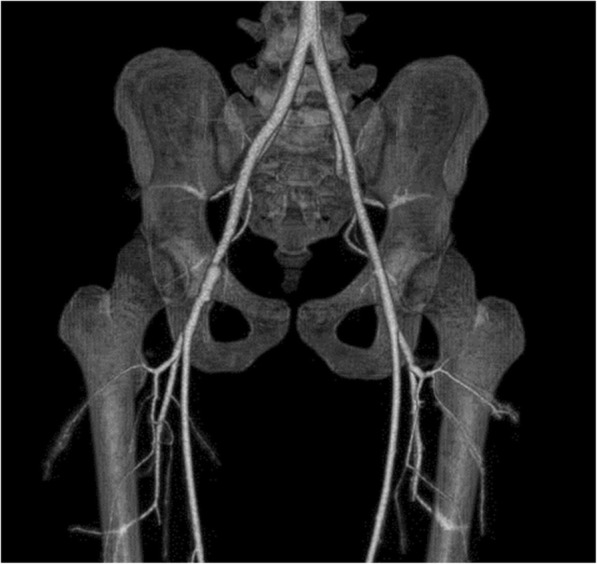
Postoperative CT angiography shows a patent right EIA and CFA and a successful repair. CT, computed tomography; EIA, external iliac artery; CFA, common femoral artery

Although the thrombus occlusion was seen in the EIA and CFA segments, judging the bruise site, we considered the CFA segment to be the main injury site. However, the possibility that the injury was also extended to the EIA segment could not be ruled out. Thus, we planned to perform an open surgery and decided to include the pararectal incision and follow the extraperitoneal approach to detect the exact extent of the injury in case the injury site was more proximal than the CFA. Intraoperatively, the CFA had a dark red color extending from the site of injury immediately below the inguinal ligament distally to just before the branching of the superficial and deep femoral arteries. No pulse was felt. A longitudinal incision of the CFA revealed a thrombus in the vascular lumen. The intima was nearly absent at the CFA injury site and was dissected on both the proximal and distal sides (Fig. 2a). The thrombus on the proximal side was removed with a Fogarty catheter® (Edwards Lifesciences, Irv, CA, USA), and the dissected intima on the proximal side and distal sides was reattached to the adventitia with 6-0 monofilament sutures. It was challenging to perform end-to-end anastomosis due to the long intimal disappearance. Although the adventitia was also damaged, its strength was relatively maintained. Therefore, a patch repair of the CFA was performed with tissue from the great saphenous vein harvested from the same surgical wound (Fig. 2b). Pulsation of the right pedal artery began postoperatively, and the right ABI improved to 1.05. Contrast CT on postoperative day 7 revealed good patency from the right EIA to the CFA, and the patch repair appeared normal (Fig. 3). The patient was discharged on day 8 and has not reported occlusion symptoms, such as numbness of the lower leg.

## Discussion

**Fig. 4:**
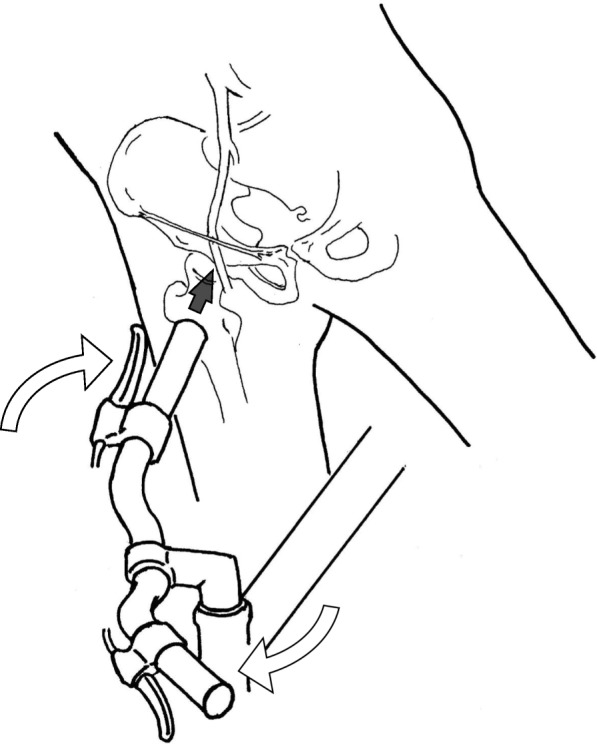
Mechanism of the bicycle handlebar injury. The bicycle collided with another bicycle, and the front wheel and handlebar of the bicycle rotated in a plane perpendicular to that of the falling rider. The point of impact was the handlebar end. The CFA and EIA are both vulnerable to this impact because they pass over the superior pubic ramus and femoral head. CFA, common femoral artery; EIA, external iliac artery

Vascular injuries often accompany bone fractures caused by blunt trauma and are rare without a concomitant bone fracture. Deutsch et al. reported the occlusion of the EIA and CFA caused by blunt trauma to the groin by motor-scooter handlebar injury, referring to it as “motor-scooter handlebar syndrome” [1]. To the best of our knowledge, in the past 20 years (1999–2018), there have been 38 similar reports of injuries of the EIA, CFA, or common iliac artery caused by blunt trauma [2–28]. The injuries were caused by handlebar trauma from bicycles [2, 3, 5–7, 11, 15, 20, 21, 24, 26], motorcycles [8, 18, 25], or all-terrain vehicles [13]; by other traffic accidents [5, 9, 10, 27]; by falling from a height [5, 22]; by being hit by a tennis ball [14]; and by blowing or compression of hard objects [5, 12, 19, 23, 28]. A rare case of CFA and vein avulsion from a hip hyperextension and abduction was also reported [17]. Seatbelt injury was reportedly a cause of blunt traumatic arterial occlusion, thereby indicating that seatbelt injuries might occur due to similar mechanisms [5, 27]. Most cases involved young adults or teenagers, and the mechanism has been described below [6]. The front wheel and handlebar of the motorcycle or bicycle rotate in a plane perpendicular to that of the falling rider, and the point of impact is with the handlebar end (Fig. 4). The femoral artery is relatively immobile because of tethering by arterial branches, periadventitial connective tissue, and the femoral sheath. Consequently, the inguinal portion the CFA is vulnerable to compression against the superior pubic ramus by the handlebar end. Regardless of the pathology, it is believed that the lesion begins as a subintimal dissection or inner circumferential intimal fracture. The thrombosis that develops may progress to complete occlusion [22]. In our patient, the thrombosis appeared to have developed because of localized intimal damage and dissection. Interestingly, the intima at the site of injury had disappeared around the entire circumference for several centimeters. It was presumed that the damaged intima had necrotized or retracted because of complete circumferential dissection. In the case of traumatic injury, the adventitia may elongate leading to a damaged intima.

Although arterial occlusion following blunt trauma is generally treated by open surgery, successful endovascular treatment has also been reported [19, 29–31]. However, the long-term outcomes of endovascular stent placement are not known. In growing adolescents and younger children, endovascular treatment could possibly lead to symptomatic occlusion of the stent (acute ischemia, claudication, and lower limb length discrepancies) due to increased caliber of the treated artery [30]. Although the patient in the present case was almost an adult, endovascular treatment was not indicated because the main injury site was CFA, which was an inappropriate zone for stenting. Thromboendarterectomy with graft interposition or patch repair and bypass grafting are standard procedures and may involve either synthetic or autologous grafts [1–18, 20–24, 26–28]. Autologous grafts are frequently preferred because the long-term patency of synthetic grafts in children is not known. Successful use of synthetic grafts in younger children has been reported [6], but it is unclear whether synthetic or autologous grafts are better. In the present case, the patient’s body size was almost the same as that of an adult. We could use a synthetic graft considering the body size but did not use it due to unclear long-term patency in such a young patient. Bypass grafting using the ipsilateral great saphenous vein is possible with the harvest of the graft from the same incisional wound, but the graft diameter is small compared with that of the CFA or EIA. The hypogastric artery is sometimes used as a large diameter graft; however, the ipsilateral hypogastric artery may be the source of collateral circulation [6]. Harvesting is complicated, and the available length is limited. In our patient, CT angiography revealed that the occlusion extended from the EIA to the CFA and that the vascular damage may have spread to the proximal side of the EIA. It might have been difficult to repair such a long lesion with an autologous vein patch. The great saphenous vein may have been inappropriate for bypass grafting because of its small diameter and the hypogastric artery for its inadequate length. Consequently, bypass grafting using a synthetic graft of suitable diameter and length might have been needed. Fortunately, the injury was confined to the CFA, thereby allowing thromboendarterectomy, patch repair using the great saphenous vein patch, and improved blood flow in the injured limb. Usually, end-to-end anastomosis is performed after artery repair if the disappearance of the intima is short. However, in our case, the long disappearance of the intima made end-to-end anastomosis challenging. Thus, we performed only patch repair considering that the adventitia was strong enough to prevent the formation of a late pseudoaneurysm. It is important to plan the surgery on the basis of the individual case characteristics.

Although prompt diagnosis is important, it may be delayed because adolescents and younger children have rapid development of collateral circulation [8]. If claudication symptoms do not appear soon after injury, vascular damage may be overlooked. In our patient, contrast CT for the evaluation of bone fracture or active bleeding led to the early detection of arterial occlusion. When examining trauma of this type, it is important to consider the differential diagnosis with the evaluation of the arterial blood flow by palpation, Doppler, ABI, duplex scan, or even contrast CT.

## Conclusions

Motor-scooter handlebar syndrome is a rare complication of traumatic injury that causes claudication and even limb length discrepancies of the lower limbs in growing children. The presence of vascular injury should be considered when patients have groin or lower abdomen injuries following impact with handlebars or similar hard objects. Surgical revascularization is important to save the ischemic limb. Surgery should be planned considering the age of the patient, preoperative imaging, and intraoperative findings.

## Abbreviations

EIA: External iliac artery
CFA: Common femoral artery
CT: Computed tomography
ABI: Ankle–brachial index
